# Professional training and case-load mix within a community mental health team

**DOI:** 10.1192/bjo.2021.603

**Published:** 2021-06-18

**Authors:** Richard Walsh, Rebecca Fahy, Ala Abdelgadir, Elizabeth Walsh, Sonn Patel

**Affiliations:** 1 School of Medicine, University College Dublin; 2 Semmelweis University; 3 Galway University Hospital

## Abstract

**Aims:**

Community Mental Health Teams (CMHTS) are now the cornerstone of modern mental health care and play a central role in assessment, diagnosis and care coordination. CMHTs vary widely in their service provision and composition. Within teams there is latitude for variation of professional roles but the extent to which different disciplines undertake generic and profession-specific work is poorly defined. This cross-sectional study aims to establish how professional training influences the distribution of case-load mix within a general adult CMHT

**Method:**

The GR1 CMHT provides care to a mixed urban/rural population of 25,000 in Galway city and Connemara. A review was conducted of multi-disciplinary case notes for all patients actively registered with the team for a period of one year. Name, age, gender, whether referred or admitted in the past year, medication and day hospital attendance were recorded. Clinical diagnoses were recorded but, where missing, verified with a relevant team member. The team consultant reviewed and verified the 1CD-10 primary clinical diagnosis for all patients. Evidence of clinical input by multidisciplinary team members was recorded from clinical files with the final electronic database being checked by each professional for accuracy. We examined any input over the past year rather than

frequency of input. Patient characteristics and diagnosis by professional discipline were examined using descriptive statistics.

**Result:**

Of a total of 246 patients registered to the team, 37.8% (N = 93) saw one, 34.6% (N = 85) saw two and 24.4% (N = 60) saw 3 or more team members. Of those who saw three or more team members, psychotic disorders represented the majority diagnoses (40%, N = 24) followed by personality disorders (25%, N = 15) and affective disorders (15%, N = 9). Patients were most commonly seen by a doctor (91.5%, N = 225) followed by community mental health nurses (CMHNs) (52.8%, N = 130). Doctors saw 85% or more of all patients grouped by ICD-10 diagnoses. The majority of social work and occupational therapy case-mix comprised psychotic disorders (SW = 44.2%, OT = 34.2%) followed by personality disorders (SW = 25.6%, OT = 23.7%). Of psychology case-mix, the highest was personality

disorders at 41.6% (N = 13) followed by anxiety and related disorders at 25% (N = 8). CMHN case-mix was highest for psychotic disorders at 44.6% (N = 58) followed by 21.5% mood disorders (N = 28).

**Conclusion:**

This cross sectional survey informs how we currently target our specialist resources. We will now develop this to include frequency of contact to inform resource allocation and skill mix.

